# Potentiating the Efficacy of Molecular Targeted Therapy for Hepatocellular Carcinoma by Inhibiting the Insulin-Like Growth Factor Pathway

**DOI:** 10.1371/journal.pone.0066589

**Published:** 2013-06-20

**Authors:** Da-Liang Ou, Bin-Shyun Lee, Ya-Chi Chang, Liang-In Lin, Jun-Yang Liou, Chiun Hsu, Ann-Lii Cheng

**Affiliations:** 1 Graduate Institute of Oncology, College of Medicine, National Taiwan University, Taipei, Taiwan; 2 National Center of Excellence for Clinical Trial and Research, National Taiwan University Hospital, Taipei, Taiwan; 3 Department of Oncology, National Taiwan University Hospital, Taipei, Taiwan; 4 Department of Clinical Laboratory Sciences and Medical Biotechnology, College of Medicine, National Taiwan University, Taipei, Taiwan; 5 Institute of Cellular and System Medicine, National Health Research Institutes, Zhunan, Miaoli County, Taiwan; 6 Department of Internal Medicine, National Taiwan University Hospital, Taipei, Taiwan; 7 Graduate Institute of Toxicology, College of Medicine, National Taiwan University, Taipei, Taiwan; Bauer Research Foundation, United States of America

## Abstract

Insulin-like growth factor (IGF) signaling pathway is an important regulatory mechanism of tumorigenesis and drug resistance in many cancers. The present study explored the potential synergistic effects between IGF receptor (IGFR) inhibition and other molecular targeted agents (MTA) in HCC cells. HCC cell lines (Hep3B, PLC5, and SK-Hep1) and HUVECs were tested. The MTA tested included sorafenib, sunitinib, and the IGFR kinase inhibitor NVP-AEW541. The potential synergistic antitumor effects were tested by median dose effect analysis and apoptosis assay in vitro and by xenograft models in vivo. The activity and functional significance of pertinent signaling pathways and expression of apoptosis-related proteins were measured by RNA interference and Western blotting. We found that IGF can activate IGFR and downstream AKT signaling activities in all the HCC cells tested, but the growth-stimulating effect of IGF was most prominent in Hep3B cells. NVP-AEW541 can abrogate IGF-induced activation of IGFR and AKT signaling in HCC cells. IGF can increase the resistance of HCC cells to sunitinib. The apoptosis-inducing effects of sunitinib, but not sorafenib, were enhanced when IGFR signaling activity was inhibited by NVP-AEW541 or IGFR knockdown. Chk2 kinase activation was found contributory to the synergistic anti-tumor effects between sunitinib and IGFR inhibition. Our data indicate that the apoptosis-potentiating effects of IGFR inhibition for HCC may be drug-specific. Combination therapy of IGFR inhibitors with other MTA may improve the therapeutic efficacy in HCC.

## Introduction

Molecular targeted therapy, which aims at specific molecular derangements in cancer cells or their microenvironment, is currently standard treatment for patients with advanced hepatocellular carcinoma (HCC) [1]. The multi-kinase inhibitor sorafenib is the first molecular targeted agent approved for the treatment of advanced HCC because of its survival benefit demonstrated by two randomized, placebo-controlled trials [2], [3]. Combination therapy of sorafenib and other molecular targeted agents are extensively tested in both pre-clinical and clinical studies to further improve treatment efficacy for advanced HCC [1], [4], [5].

The insulin-like growth factor (IGF) signaling pathway plays important roles in HCC tumorigenesis [6], [7]. Increase in both IGF and IGF receptor (IGFR) gene expression was found in human cirrhotic liver, in HCC tissue, and in human HCC cell lines [8]–[10]. This suggested that IGF signaling may stimulate hepatocarcinogenesis via autocrine or paracrine mechanisms [11]. Up-regulation of IGF and IGFR may be induced by hepatitis B virus x protein [12], [13] and p53mt249 [14], a gain-of function mutant of p53 that is associated with HCC and aflatoxin B1 exposure. This suggested that IGF signaling is closely associated with other tumorigenic processes of HCC and may serve as a therapeutic target.

Activation of the IGF signaling pathway may increase cancer cell proliferation, stimulate aggressive tumor behavior in established cancers [15], and confer resistance of cancer cells to cytotoxic and molecular targeting therapies [16]–[18]. Inhibition of the IGF signaling pathway, on the other hand, may inhibit cancer cell proliferation and metastasis [19], [20] and increase the sensitivity of cancer cells to cytotoxic agents [21], [22]. The chemo-sensitizing effects of IGF signaling blockade have been demonstrated in many different tumor models, including HCC [23], [24]. In addition, IGF signaling pathway may also be involved in tumor-associated angiogenesis [25]. Multiple strategies targeting the IGF signaling pathway have been tested for their potential as anticancer therapies [26].

The present study sought to clarify whether inhibition of the IGF signaling pathway can enhance the efficacy of molecular targeted therapy in HCC. Effects of IGFR inhibition on the activities of IGF and downstream signaling pathways in HCC cells were determined. Potential synergistic anti-tumor activities between IGFR inhibition and other molecular targeted therapy were explored.

## Methods

### Ethics Statement

The protocol for the xenograft experiments in this study was approved by the Institutional Animal Care and Use Committee of the College of Medicine, National Taiwan University, and conformed to the criteria outlined in the Guide for the Care and Use of Laboratory Animals prepared by the National Academy of Sciences and published by the National Institutes of Health.

### Cell Culture

HCC cell lines, including Hep3B, PLC5 and SK-Hep1, were obtained from the American Type Culture Collection (ATCC). Cells were cultured in Dulbecco’s modified Eagle’s medium supplemented with 10% fetal bovine serum (FBS), 100 units/mL penicillin, and 100 µg/mL streptomycin. Primary human umbilical venous endothelial cells (HUVEC) were cultured as described before [4]. All cell lines were grown in 5% CO2 at 37°C and confirmed negative for Mycoplasma contamination by using the EZ-PCR mycoplasma test kit **(**Biological Industries Israel Beit-Haemek Ltd, Israel**)**.

### Chemicals and Antibodies

The IGFR inhibitor used in this study was a small-molecule IGFR kinase inhibitor NVP-AEW541 (Novartis), with an in vitro kinase inhibitionIC_50_ at 0.086****µM [27]. Other molecular targeted agents (MTAs) tested in this study included sorafenib (Bayer-Schering Pharma), and sunitinib (Pfizer). For in vitro experiments, the drugs were dissolved in DMSO, and the final concentration of DMSO was kept below 0.1%. For in vivo experiments, sorafenib was dissolved in Cremophor EL/95% ethanol (50∶50; Sigma-Aldrich, St. Louis, MO), NVP-AEW541 was dissolved in tartaric acid (25 mmol/L). Sunitinib was dissolved in carboxymethylcellulose solution (carboxymethylcellulose 0.5%, NaCl 1.8%, Tween 80 0.4%, and benzyl alcohol 0.9% in distilled water) and adjusted to pH 6.0. IGF1 and IGF2 were purchased from R&D Systems (Minneapolis, MN). The antibodies used for Western blotting (used at 1∶1000 dilution) and immunohistochemical staining (used at 1∶100 dilution) included p-IGF-1R, p-AKT, caspase 3, p-EGFR (Tyr1086), EGFR, p-Chk2, Chk2 (Cell Signaling Technology, Danvers, MA), IGF-1R, ERK 2, p-ERK 1/2, and PARP-1 (Santa Cruz Biotechnology, Santa Cruz, CA).

### Cell Viability and Median Effect Analysis

Cell viability was assessed using an MTT (3-(4, 5-dimethylthiazol-2-yl)-2, 5- diphenyltetrazolium bromide) assay. The IC_50_ values after drug treatment were calculated using CompuSyn software (ComboSyn, Paramus, NJ) based on the changes of absorbance measured by spectrophotometry (DTX 880; Beckman Coulter, Fullerton, CA). All the MTAs used in this study were tested in cell-free condition to confirm the lack of interference with the MTT kit reagents (see Table S1). The potential synergistic antitumor effects between different drug treatments were measured by median dose-effect analysis using the combination index (CI)-isobologram method, as described before [4].

### Apoptosis Assay

Apoptotic cell death was measured by flow cytometry (sub-G1 fraction analysis) and Western blot analysis (caspase activation, PARP cleavage).

### Western Blot Analysis and Phospho-antibody Kinase Array

Activity of IGFR and downstream signaling pathways, including the PI3K/AKT and MEK/ERK pathways, was measured by Western blot analysis. To explore the effects of IGFR inhibition on other pertinent signaling pathways, a phospho-antibody kinase array (Proteome Profiler™, R&D Systems, Minneapolis, MN), which can simultaneously measure relative levels of phosphorylation of 46 kinase phosphorylation sites, was used (see Figure S1 for the list of the phospho-proteins analyzed). Signals were visualized using a UVP Imaging System (UVP, Upland, CA) or with X-ray films. Candidate molecules were selected based on their expression levels after drug treatment and correlation with the effects of in vitro antitumor effects. The expression levels of these candidate molecules after drug treatment were confirmed by Western blot analysis.

### Gene Knockdown using siRNA

To explore the biological roles of the candidate molecules, small interfering RNA (siRNA) knockdown was used in combination with treatment by MTAs. The candidate si-Chk2 (ID: s22119), si-IGF1R-a (ID: s7211), si-IGF1R-b (ID: s7212) and scrambled nonspecific (negative-control#1) siRNA were purchased from Ambion (Austin, TX). Cells were transfected with siRNA using the siPORT NeoFx transfection reagent (Ambion) and treated with the MTAs. The treated cells were then collected for subsequent Western blot or flow cytometry analysis.

### Xenograft Tumor Growth

Tumor xenograft experiments were performed by using male BALB/c athymic (nu+/nu+) mice and Hep3B cells, which were inoculated subcutaneously at 1×10^6^cells. The mice were randomized to different treatment groups of MTAs (animal number n = 5 in each treatment group) when the tumor volume reached approximately 100 mm^3^ (volume [mm^3^] = [width]^2^× length ×0.5). MTAs treatment was administered daily by gavage. Tumor volume and body weight were recorded every 4 days. Fresh-frozen tumor samples after MTAs treatment were collected to measure the levels of pertinent proteins by Western blot. Formalin-fixed, paraffin-embedded tumor samples after drug treatment were collected for immunohistochemical analysis of pertinent protein expression and tumor angiogenesis. TUNEL assay was performed to measure the extent of tumor cell apoptosis, as described before [4].

### Statistical Analysis

All data were representative of at least three independent experiments. Quantitative data are expressed as mean ± SD. Comparisons were analyzed using the Student's *t* test and ANOVA. Significance was defined as *p*<0.05.

## Results

### Effects of IGFR Signaling Modulation on Drug Resistance of HCC Cells

The growth-inhibitory effects of the IGFR inhibitor NVP-AEW541 on HCC cells and HUVEC were determined by MTT assay. The IC_50_ of NVP-AEW541 was 2.22±0.55 µM for Hep3B, 2.78±0.67 µM for SK-Hep1, 3.27±0.50 µM for PLC5, and 5.70±0.54 µM for HUVEC. These IC_50_ levels greatly exceed the IC_50_ of IGFR inhibition (0.086 µM by in vitro kinase assay) [27]. Concentrations of NVP-AEW541 in subsequent experiments were limited to 1 µM or lower to avoid non-specific activity.

**Figure 1:**
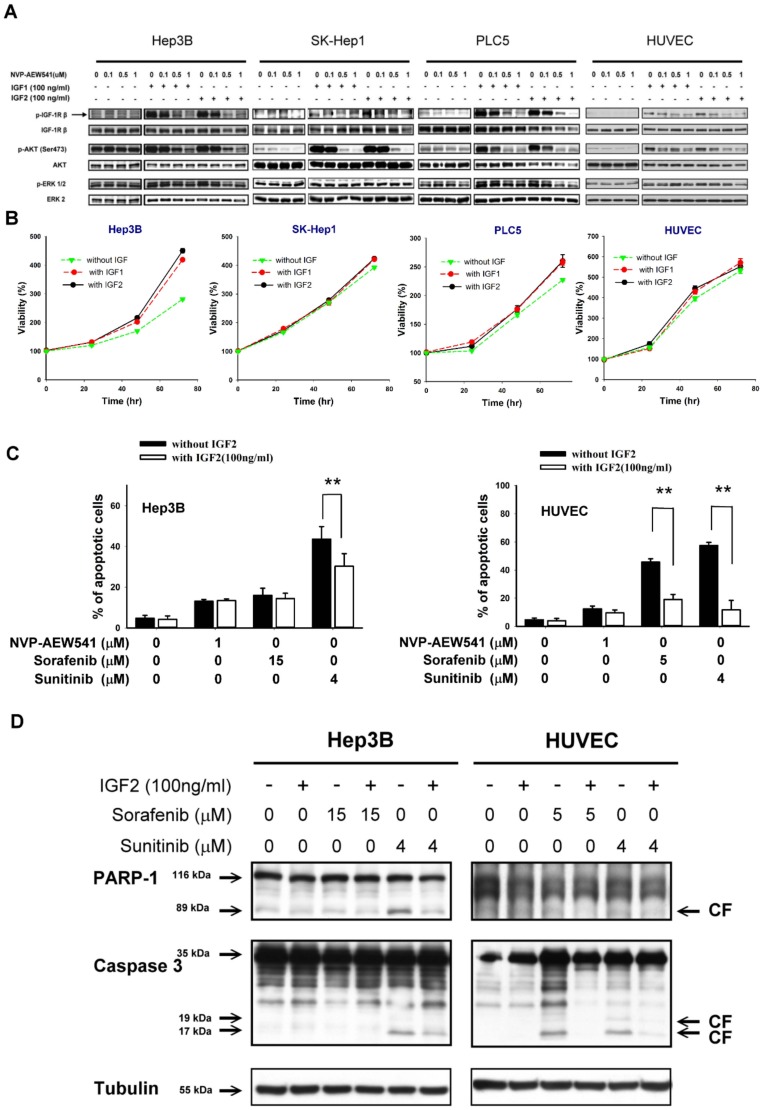
Effects of IGFR signaling modulation on drug resistance of HCC cells. (A) Addition of IGF1 or IGF2 (100 ng/ml) into the culture medium can activate IGFR and AKT signaling in all the HCC cells (Hep3B, SK-Hep1, and PLC5) and HUVEC. The activation can be inhibited by the IGFR inhibitor NVP-AEW54. (B) Growth curves of HCC cells and HUVEC with or without stimulation by IGF1 or IGF2. Cell viability was assessed by MTT assay. Points, mean (n = 3); bars, SD**.** (C) Percentage of apoptotic cells was measured by flow cytometry after 72 hours of drug treatment in Hep3B and HUVEC. **, p<0.05 compared with cells untreated with IGF2. (D) Effects of IGF stimulation on sorafenib, and sunitinib on P ARP-1 cleavage and caspase 3 activation.

To explore the possible effects of IGF in the tumor microenvironment on cell growth and drug resistance, IGF1 or IGF2 was added to the culture medium of HCC cells and HUVEC. Addition of IGF1 or IGF2 (100 ng/ml) can activate IGFR and AKT signaling in all the HCC cells tested (Hep3B, SK-Hep1, and PLC5) and HUVEC to a similar extent. This activation can be inhibited by NVP-AEW541 in a dose-dependent manner (Figure 1A). However, effects on cell growth after IGF stimulation varied, with only Hep3B cells showed a significant increase in cell growth (Figure 1B). IGF2 was used in subsequent experiments because it has been more frequently reported to be over-expressed in the liver microenvironment [7].

We hypothesized that IGF stimulation can increase resistance of HCC cells to MTAs. As shown in Figure 1, apoptosis induced by sunitinib in Hep3B cells can be reduced by IGF2, while apoptosis induced by sorafenib or NVP-AEW541 did not change significantly (Figure 1C and 1D). On the other hand, in HUVEC the apoptosis-reducing effects of IGF were seen after treatment with sunitinib or sorafenib (Figure 1C and 1D). The results suggest that the effects of IGF in tumor microenvironment on drug resistance may depend on both the cell types and the drug tested.

### Potential Synergistic Anti-tumor Effects between IGFR Inhibition and Other MTAs

**Figure 2:**
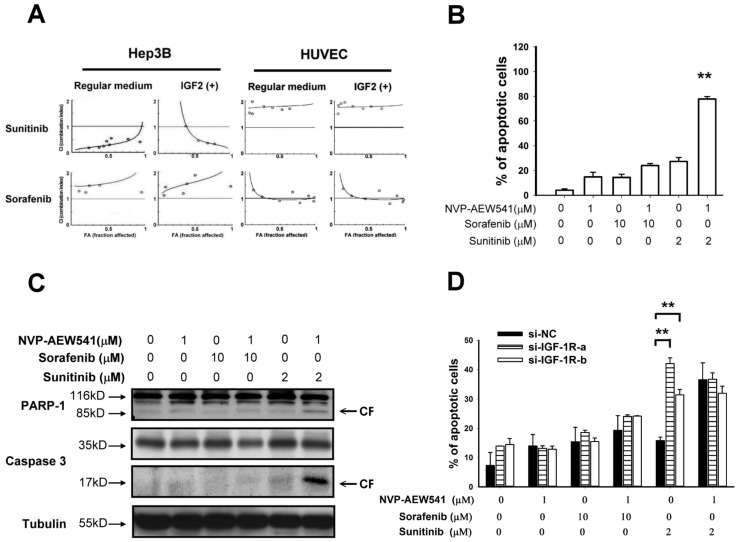
Synergistic growth-inhibitory and apoptosis-inducing effects between IGFR inhibition and other MTAs. (A) Median dose effect analysis of synergistic growth inhibitory effects. Growth inhibition was measured by MTT assay. CI was calculated using the CI-isobologram method. CI = 1, additive effect; CI <1, synergistic effect; CI >1, antagonistic effect. (B and C) Synergistic apoptosis-inducing effects between IGFR inhibition and other MTAs in Hep3B cells measured by flow cytometry (sub-G1 fraction analysis) (B) and by PARP-1 cleavage and caspase 3 activation (C). **, p<0.05 compared with cells not treated with the IGFR inhibitor NVP-AEW541. (D) IGFR inhibition by siRNA knockdown enhanced the apoptosis-inducing effects of sunitinib, but not sorafenib or NVP-AEW541, in Hep3B cells.

In the next step we explored the potential synergistic anti-tumor effects between IGFR inhibition and other MTAs. Synergistic growth-inhibitory effects in Hep3B cells were found when the IGFR inhibitor NVP-AEW541 was combined with sunitinib but not with sorafenib. This synergistic effect was similar with or without IGF stimulation in the medium. By contrast, no significant synergistic growth-inhibitory effects were seen in HUVEC (Figure 2A).The synergistic effects between IGFR inhibition and sunitinib in Hep3B cells resulted from increased induction of apoptosis (Figure 2B and 2C). Knockdown of IGFR by siRNA can enhance the apoptosis-inducing effects of sunitinib in Hep3B cells to a similar extent as NVP-AEW541 did, supporting the roles of IGFR signaling in modulation of drug resistance in HCC cells (Figure 2D).

### p-Chk2 is a Possible Mediator of Anti-tumor Synergy between IGFR Inhibition and Sunitinib

**Figure 3:**
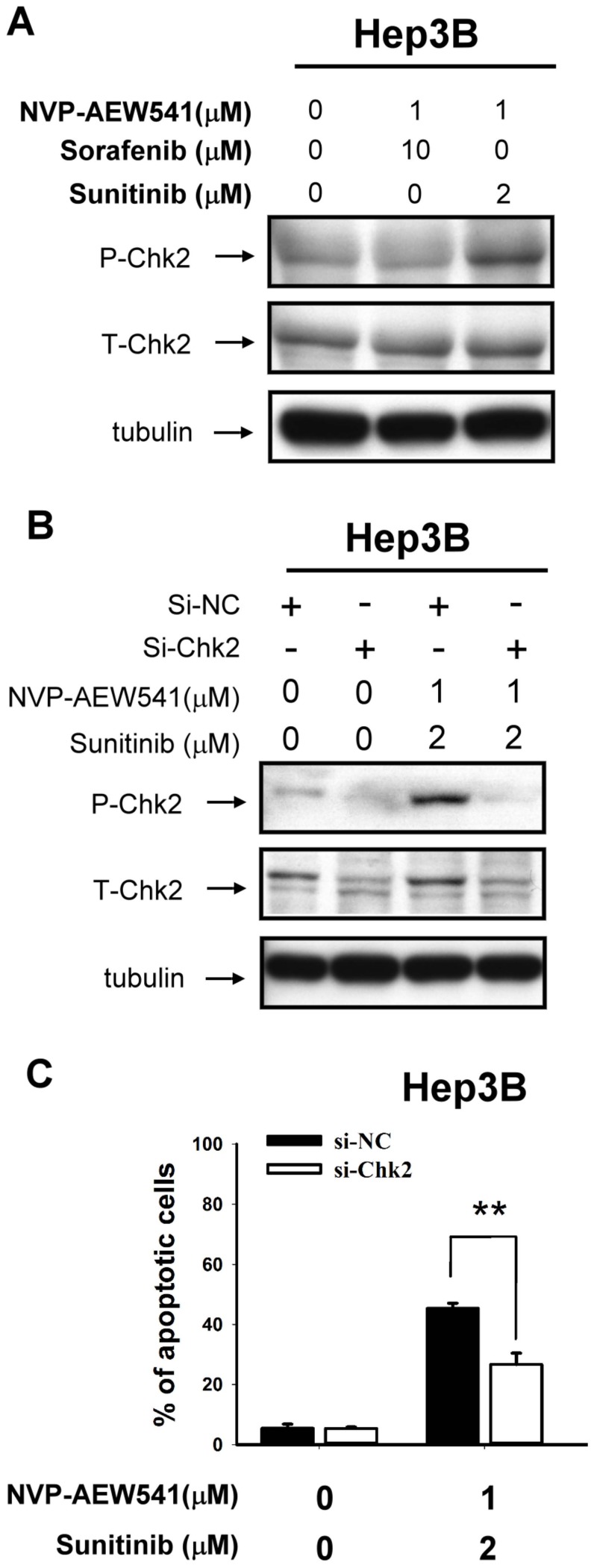
p-Chk2 is a possible mediator of anti-tumor synergy between IGFR inhibition and sunitinib. (A) Effects between NVP-AEW541, sorafenib, and sunitinib on apoptosis-related proteins in HCC cells. Hep3B cells were treated with NVP-AEW541, sorafenib, and sunitinib at the indicated concentrations for 48 h. Whole-cell lysates were subjected to Western blotting. (B and C) Hep3B cells were transfected with si-Chk2 or scrambled siRNA for 24 hours, and then treated with the indicated drugs. Whole-cell lysates were collected for Western blotting after 48 h drug treatment. The percentages of apoptotic cells were measured by flow cytometry after 72 h drug treatment. **, p<0.01.

Although AKT signaling is considered an important mechanism of drug resistance in cancer cells, our data indicated that modulation of AKT signaling activity did not change the anti-tumor synergy between NVP-AEW541 and sunitinib (see Figure S2). Results from screening of phospho-protein expression after drug treatment by the phospho-protein array suggested that Chk2 kinase may be a possible mediator of the synergy between NVP-AEW541 and sunitinib in Hep3B cells (Figure S3). Inhibition of IGFR signaling by NVP-AEW541 or si-RNA knockdown can enhance the up-regulation of Chk2 phosphorylation by sunitinib (Figure 3A and Figure S4), and knockdown of Chk2 expression can reduce apoptosis induced by NVP-AEW541 plus sunitinib (Figure 3B, 3C and Figure S5). The above data supports the roles of Chk2 as a downstream mediator of anti-tumor synergy between IGFR inhibition and sunitinib.

### In vivo Anti-tumor Synergy between IGFR Inhibition and Other MTAs

**Figure 4:**
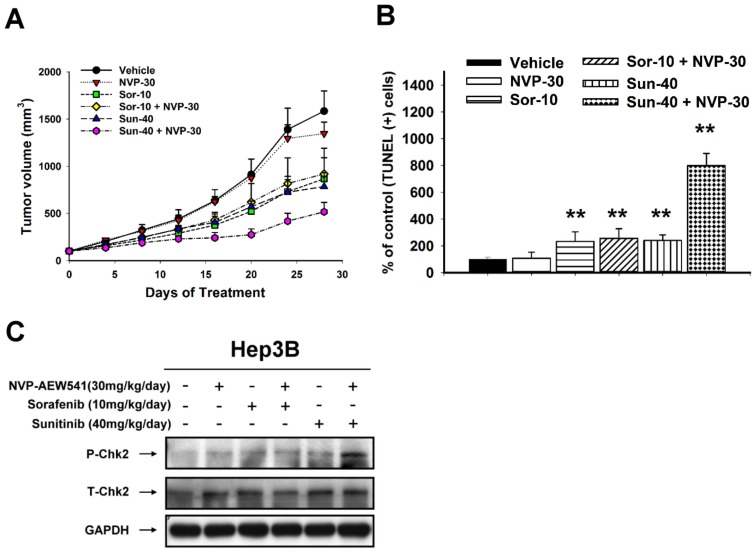
In vivo anti-tumor synergy between IGFR inhibition and other MTAs. Hep3B cells were injected subcutaneously into male BALB/c athymic nude mice. Mice were treated daily by gavage as indicated (NVP-30, NVP-AEW541 30 mg/kg/day; Sor-10, sorafenib 10 mg/kg/day; Sun-40, sunitinib 40 mg/kg/day, animal number n = 5 in each treatment group). Difference in (A) tumor growth; (B) tumor cell apoptosis (TUNEL assay).**, P<0.01, compared with control (vehicle treatment). (C) Difference in Chk2 phosphorylation measured by Western blotting.

The anti-tumor synergy between NVP-AEW541 and sunitinib was also confirmed by xenograft experiments (Figure 4). Combination of NVP-AEW541 with sunitinib inhibited tumor growth significantly better than single-agent treatment. By contrast, NVP-AEW541 did not enhance the efficacy of sorafenib (Figure 4A). NVP-AEW541-sunitinib combination induced more tumor cell apoptosis than single-agent treatment (Figure 4B), while tumor angiogenesis did not differ significantly (see Figure S6). The in vivo effects on Chk2 phosphorylation were consistent with changes seen in in vitro studies (Figure 4C).

## Discussion

This study sought to explore the roles of IGF signaling pathway in molecular targeted therapy for HCC. Our data suggest that IGF can increase resistance of HCC cells and HUVEC to molecular targeted therapy. The small-molecule IGFR inhibitor NVP-AEW541 can enhance apoptosis of HCC cells induced by selected MTAs, such as sunitinib. The apoptosis-enhancing effects of IGFR inhibition may involve different downstream signaling pathways, including Chk2, when IGFR inhibitors are combined with sunitinib.

Although inhibition of IGF signaling pathway has shown promising anti-tumor activity in pre-clinical studies, clinical trials of IGFR inhibitors, including neutralizing antibodies to IGF, receptor-blocking antibodies, and small-molecule IGFR kinase inhibitors, have only limited success [27]. Our data indicate that NVP-AEW541 has minimal anti-tumor activity at concentration specific for IGFR inhibition (<1 µM). This is consistent with the negative results of clinical trials of monoclonal antibodies targeting IGFR in patients with advanced HCC [28], [29]. Therefore, IGFR inhibitors may play a more important role in combination therapy rather than single-agent therapy in HCC.

An important issue in optimizing treatment targeting the IGFR signaling pathway is identification of pertinent predictive biomarkers for treatment efficacy. Genetic aberrations of IGFR pathway in HCC tumor cells are rare [30], [31]. Therefore, signaling activities of IGFR and downstream pathways are usually evaluated by immunohistochemical studies of tumor tissue. However, the lack of standardized tissue processing and instability of phospho-protein levels significantly undermines the reliability of this approach [32], [33]. Future clinical trials should address molecular sub-classification of HCC and patient enrichment based on biomarker profiles to improve the chance of success.

Our data indicate that Chk2, a critical regulator of DNA damage response, may contribute to resistance to selected MTAs. Chk2 activation can lead to cell cycle arrest, senescence, or apoptosis, depending on the cellular genetic context and types and intensity of DNA damage [34]. Inhibition of Chk2 activation can enhance the anti-cancer efficacy of DNA-damaging agents [35]. Recent pre-clinical studies indicate that MTAs play important roles in regulation of DNA damage response in glioma cells, which may contribute to resistance to DNA-damaging therapy [36]. The roles of Chk2 activation and other regulators of DNA damage response in resistance of cancer cells to molecular targeted therapy warrant further investigation.

Resistance of cancers to IGFR inhibitors has been extensively studied. An important mechanism of resistance is the compensatory activation of related signaling pathways. The PI3K/AKT and the MEK/ERK pathways are the most extensively studied. AKT signaling has been demonstrated to be a critical mechanism of drug resistance in HCC cells [37], [38]. However, AKT may be activated by multiple upstream pathways, and inhibition by specific upstream MTAs may result in compensatory activation via other pathways. Recent pre-clinical studies indicated that vertical blockade of the PI3K/AKT/mTOR pathway, i.e., combination of agents targeting more than one molecule in a signaling pathway, can produce synergistic anti-cancer efficacy with acceptable toxicity [39], [40]. This concept warrants further clinical exploration for the treatment of HCC.

Over-expression of IGF-2 in the tumor microenvironment was hypothesized as another potential mechanism of resistance [26]. This hypothesis was supported by our findings that IGF-2 can antagonize apoptosis induced by MTAs in HCC cells. In addition, increased serum IGF-2 was found to be associated with poorer treatment efficacy of sorafenib in advanced HCC patients. Binding of IGF to IGFR induced anti-apoptotic effects, whereas un-occupied IGFR induced pro-apoptotic effects independent of IGFR kinase activation, a phenomenon known as ‘dependence receptor’ [41]. The above data indicate that increased IGF levels in tumor microenvironment may play important roles in determining clinical efficacy of molecular targeted therapy for HCC. Expression of insulin receptor on cancer cells, which can mediate IGF signaling and maintain the malignant phenotypes, may also contribute to resistance to IGFR inhibition [26], [42].

In conclusion, our data suggest that the IGFR signaling pathway plays important roles in resistance of HCC to MTAs. Multiple downstream signaling pathways may contribute to the resistance phenotypes. Further dissection of the interactions between different signaling pathways is needed to identify the optimal combination strategy for future clinical trials.

## Supporting Information

Figure S1 **The list of kinases measured by the Human phospho-antibody kinase array kit (Proteome Profiler™, R&D Systems, Minneapolis, MN).**
(DOCX)

Figure S2 **Modulation of AKT signaling activity did not correlate with the anti-tumor synergy between NVP-AEW541 and sunitinib.**
(DOCX)

Figure S3 **Screening of phosphor-protein expression in HCC cells after treatment with molecular targeted agents.**
(DOCX)

Figure S4 **Increased Chk2 phosphorylation by sunitinib plus IGFR inhibition.**
(DOCX)

Figure S5 **Increased Chk2 phosphorylation contributes to the anti-tumor synergy in SK-hep1 cells between sunitinib and IGFR inhibition.**
(DOCX)

Figure S6 **The potential anti-angiogenic effects of IGFR inhibition and other MTAs.**
(DOCX)

Table S1 Confirmation of the lack of interference with the MTT kit reagents.
(DOCX)
