# Supplementary material for: Potentiating the Efficacy of Molecular Targeted Therapy for Hepatocellular Carcinoma by Inhibiting the Insulin-Like Growth Factor Pathway
Source: PLoS One. 2013 Jun 20;8(6):e66589. doi: 10.1371/journal.pone.0066589 (PMC3688529; doi:10.1371/journal.pone.0066589)

Table S1

Confirmation of the lack of interference with the MTT kit reagents. The test compounds were pre-diluted and transferred to 96-well plates by automated liquid handling. 96-well plates were incubated with test compounds for 72 hrs, after which 50 µg/mL of 3-(4,5-Dimethyl-2-thiazolyl)-2,5-dipheny​l-2H-tetrazolium bromide (MTT) was added. After incubation for 4 hrs at 37°C, the medium was discarded and 150 µL of dimethylsulfoxide (DMSO) was added into each well to dissolve the formazan crystals. The absorbance was determined at 540 nm by spectrophometry (DTX 880; Beckman Coulter, Fullerton, CA). As shown in the following table, all the OD values were negligible.

Figure S1.

The list of kinases measured by the Human phospho-antibody kinase array kit (Proteome Profiler^TM^, R&D Systems, Minneapolis, MN)


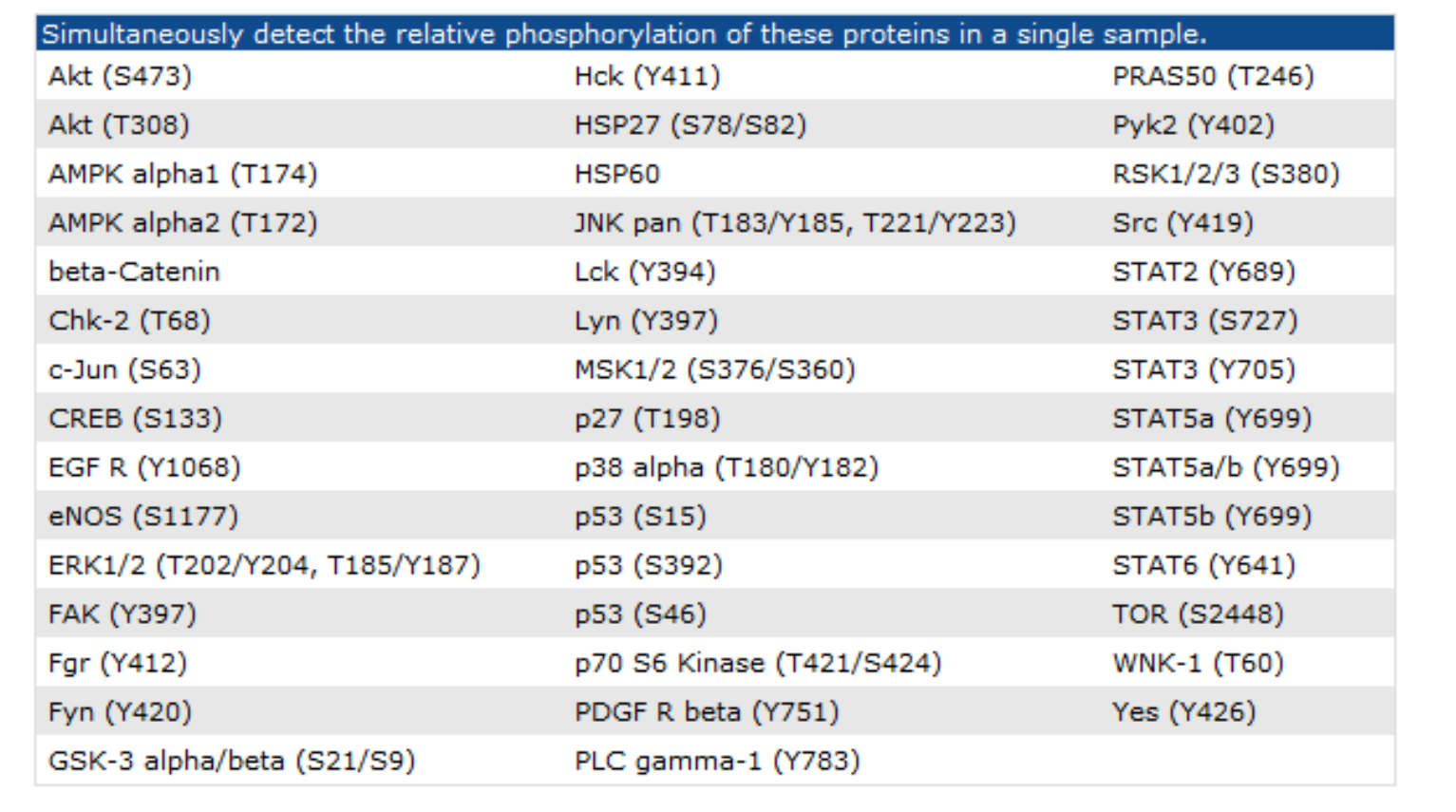


Figure S2.

Modulation of AKT signaling activity did not correlate with the anti-tumor synergy between NVP-AEW541 and sunitinib.

1. Hep3B cells were kept under starvation for 12 hours. Then, cells were treated by IGF2 (100 ng/ml) and different targeted agents for 2 hours. Whole-cell lysates were then analyzed by Western blotting. Although IGF-induced activation of IGFR, AKT, and ERK signaling activities can be suppressed to various extents, the extent of inhibition did not correlate with the anti-tumor synergy between NVP-AEW541 and sunitinib.

(B and C) Hep3B cells were transfected with constitutively active Akt1 construct or an empty pUSEamp vector, and then the cells were treated with the indicated drugs.The results indicate that constitutively active AKT cannot rescue the cells from apoptosis induced by NVP-AEW541 plus sunitinib. The vector constructs, purchased from Millipore-Upstate (Temecula, CA), were transiently transfected into Hep3B cells using Lipofectamine 2000 (Invitrogen). Whole-cell lysates were collected after 24-hour drug treatment for Western blotting. Percentage of apoptotic cells was measured after 72-hour drug treatment by flow cytometry.


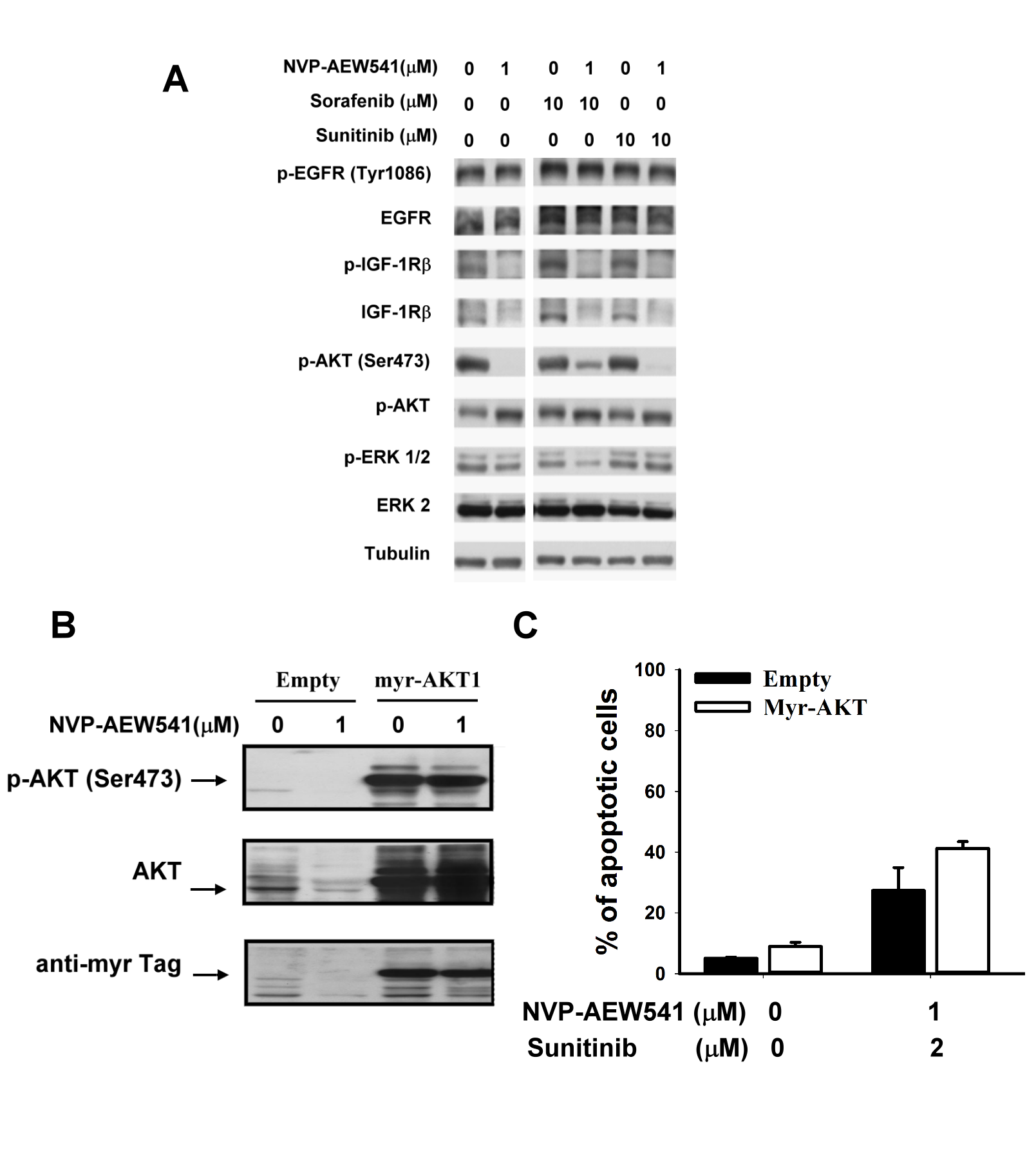


Figure S3.

Screening of phosphor-protein expression in HCC cells after treatment with molecular targeted agents.Hep3B cells were treated with differentdrugs at the indicated concentrations for 48 hours. Whole-cell lysates were incubated with membranes of the Human phospho-antibody kinase array kit (Proteome Profiler^TM^, R&D Systems, Minneapolis, MN) according to the manufacturer’s instructions.


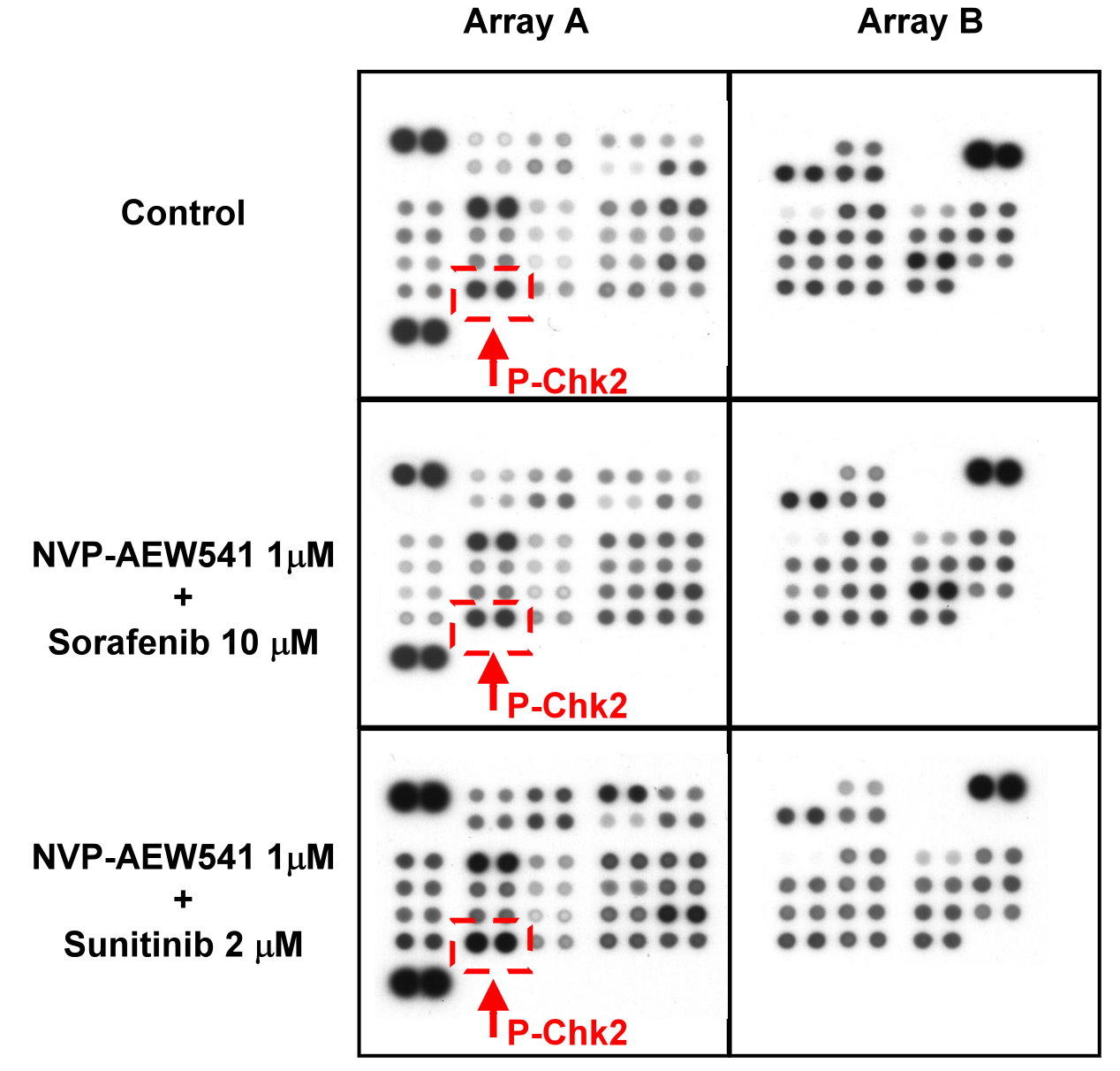


Figure S4.

Increased Chk2 phosphorylation by sunitinib plus IGFR inhibition.

(A) Hep3B cells were transfected with si-IGF1R-a, si-IGF1R-b or scrambled siRNA for 24 hours, and then treated with the indicated drugs. Whole-cell lysates were collected for Western blotting after 48 h drug treatment. (B) Effects between NVP-AEW541 and sorafenib or sunitinib on Chk2 phosphorylation in SK-Hep1 cells. SK-Hep1 cells were treated with NVP-AEW541 and sorafenib or sunitinib at the indicated concentrations for 48 h. Whole-cell lysates were subjected to Western blotting.


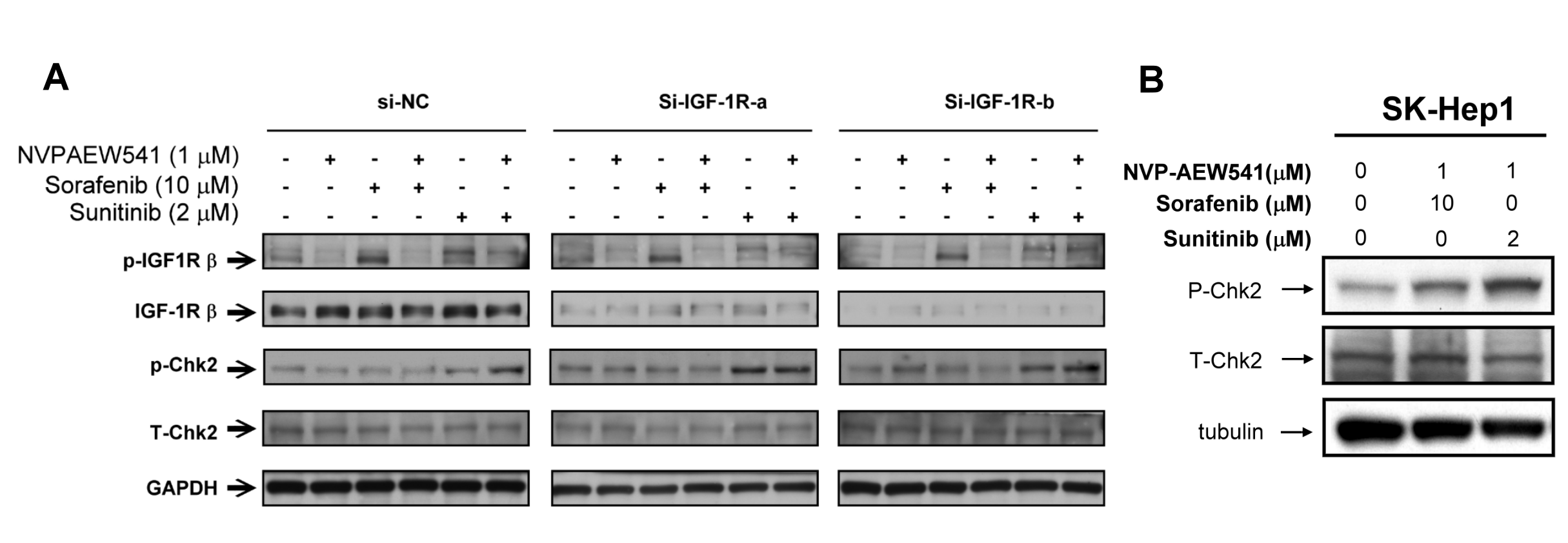


Figure S5.

Increased Chk2 phosphorylation contributes to the anti-tumor synergy in SK-hep1 cells between sunitinib and IGFR inhibition.

(A) Median dose effect analysis of synergistic growth inhibitory effects. Growth inhibition was measured by MTT assay. CI was calculated using the CI-isobologram method. CI = 1, additive effect; CI < 1, synergistic effect; CI > 1, antagonistic effect.(B and C) SK-Hep1 cells were transfected with si-Chk2 or scrambled siRNA for 24 hours, and then treated with the indicated drugs. Whole-cell lysates were collected for Western blotting after 48 h drug treatment. The percentages of apoptotic cells were measured by flow cytometry after 72 h drug treatment. **, p < 0.01.


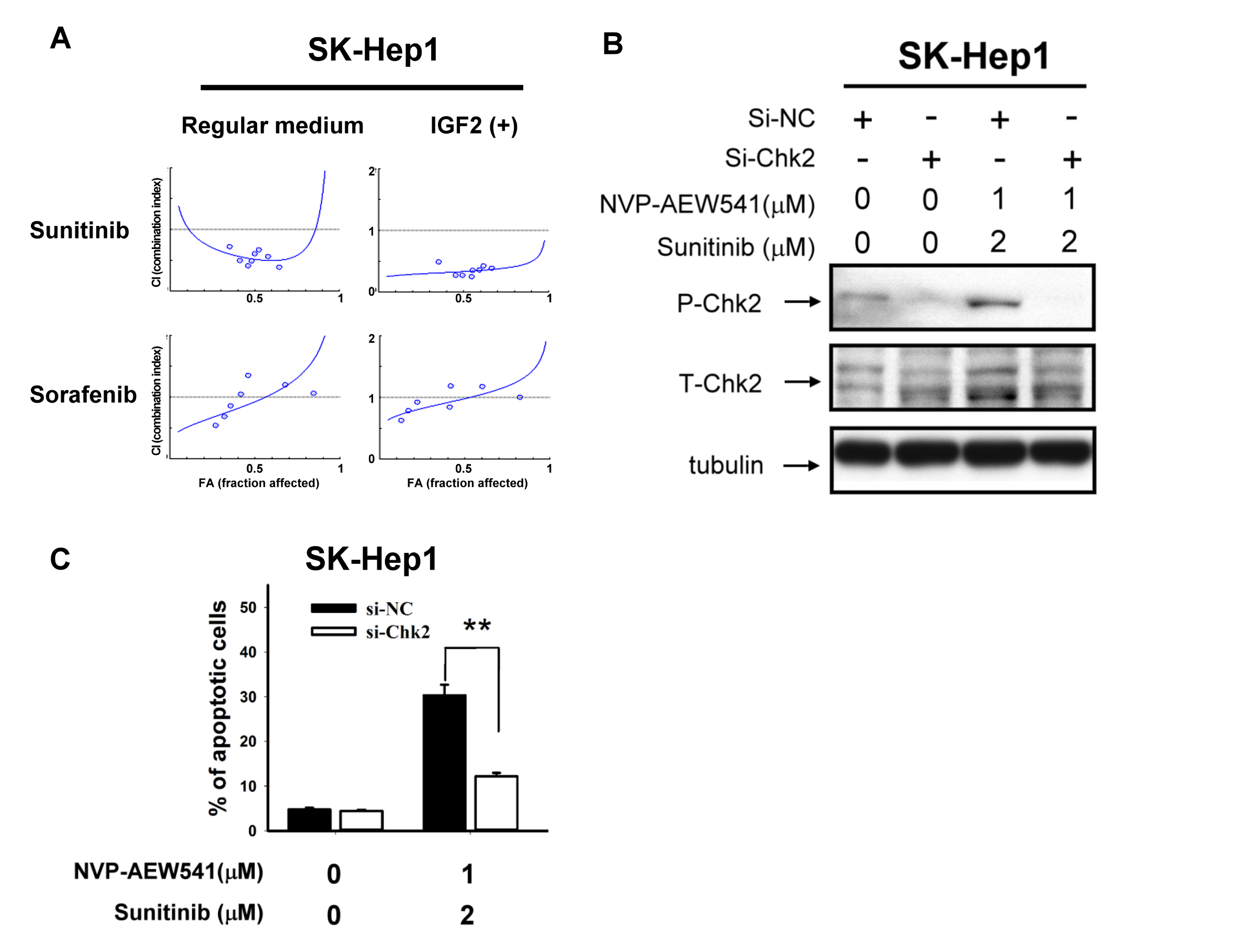


Figure S6.

The potential anti-angiogenic effects of IGFR inhibition and other MTAs. Hep3B cells were injected subcutaneously into male BALB/c athymic nude mice. Mice were treated daily by gavage as indicated (NVP-30, NVP-AEW541 30 mg/kg/day; Sor-10, sorafenib 10 mg/kg/day; Sun-40, sunitinib 40 mg/kg/day). Tumor microvessel density (MVD) was measured by CD31 immunohistochemical staining (animal number n = 4 in each group). **, P < 0.01, compared with the control (vehicle-treated) group.


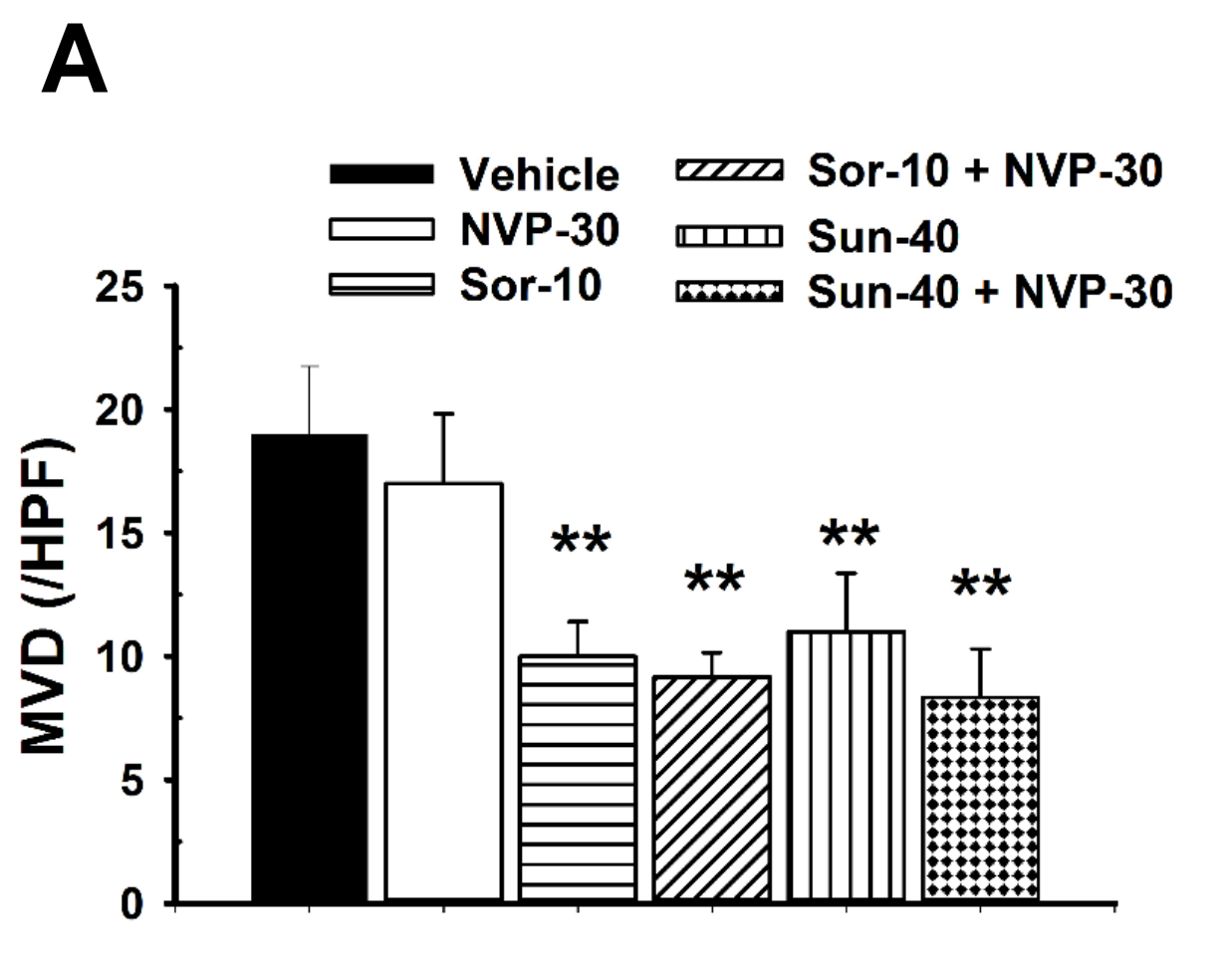

Supplement: Figure S1 — The list of kinases measured by the Human phospho-antibody kinase array kit (Proteome Profiler™, R&D Systems, Minneapolis, MN). (DOCX) [file pone.0066589.s001.docx]
