# Supplementary material for: Potentiating the Efficacy of Molecular Targeted Therapy for Hepatocellular Carcinoma by Inhibiting the Insulin-Like Growth Factor Pathway
Source: PLoS One. 2013 Jun 20;8(6):e66589. doi: 10.1371/journal.pone.0066589 (PMC3688529; doi:10.1371/journal.pone.0066589)

Figure S3.

Screening of phosphor-protein expression in HCC cells after treatment with molecular targeted agents.Hep3B cells were treated with differentdrugs at the indicated concentrations for 48 hours. Whole-cell lysates were incubated with membranes of the Human phospho-antibody kinase array kit (Proteome Profiler^TM^, R&D Systems, Minneapolis, MN) according to the manufacturer’s instructions.


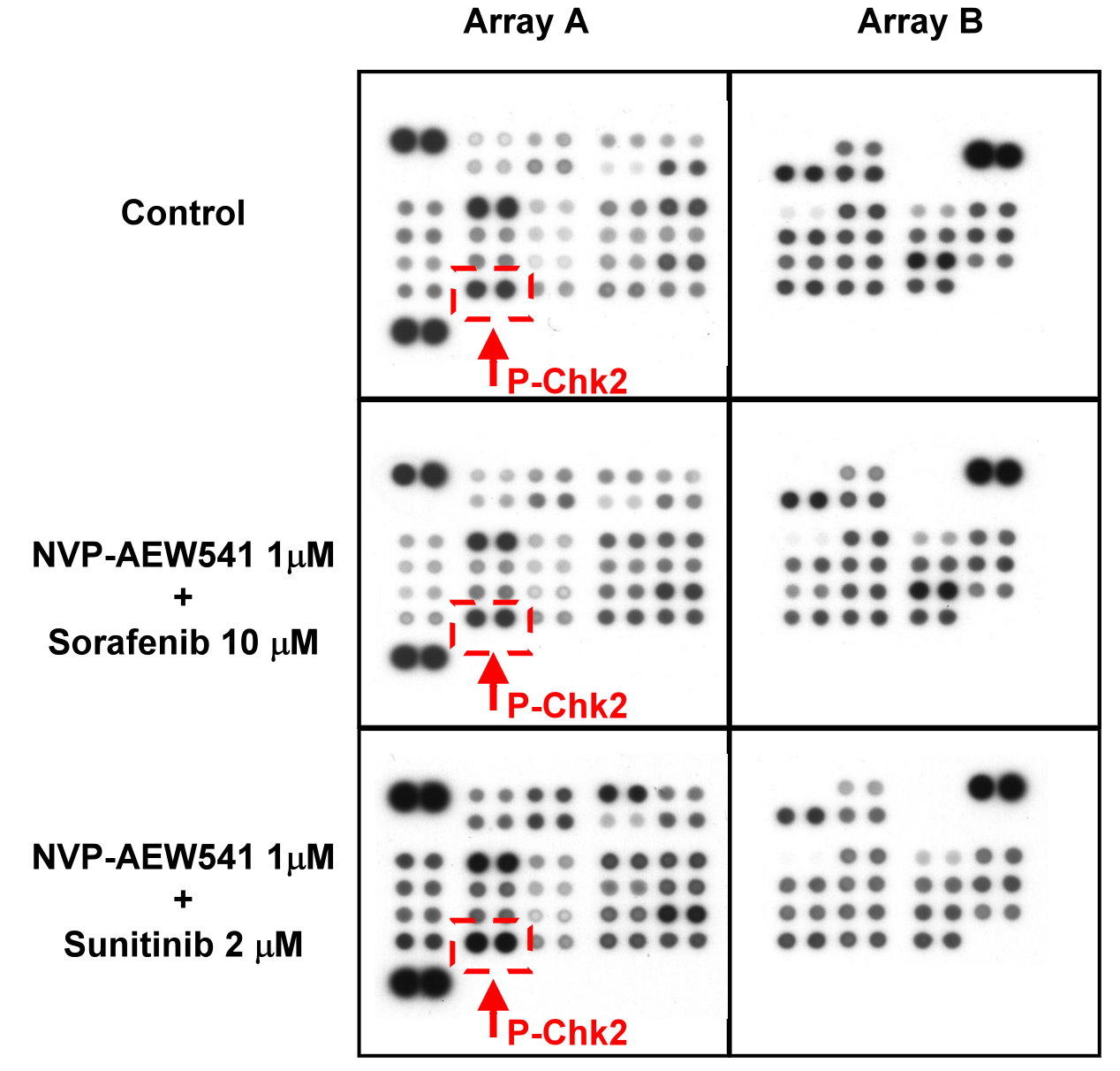

Supplement: Figure S3 — Screening of phosphor-protein expression in HCC cells after treatment with molecular targeted agents. (DOCX) [file pone.0066589.s003.docx]
