# Supplementary material for: Potentiating the Efficacy of Molecular Targeted Therapy for Hepatocellular Carcinoma by Inhibiting the Insulin-Like Growth Factor Pathway
Source: PLoS One. 2013 Jun 20;8(6):e66589. doi: 10.1371/journal.pone.0066589 (PMC3688529; doi:10.1371/journal.pone.0066589)

Figure S4.

Increased Chk2 phosphorylation by sunitinib plus IGFR inhibition.

(A) Hep3B cells were transfected with si-IGF1R-a, si-IGF1R-b or scrambled siRNA for 24 hours, and then treated with the indicated drugs. Whole-cell lysates were collected for Western blotting after 48 h drug treatment. (B) Effects between NVP-AEW541 and sorafenib or sunitinib on Chk2 phosphorylation in SK-Hep1 cells. SK-Hep1 cells were treated with NVP-AEW541 and sorafenib or sunitinib at the indicated concentrations for 48 h. Whole-cell lysates were subjected to Western blotting.


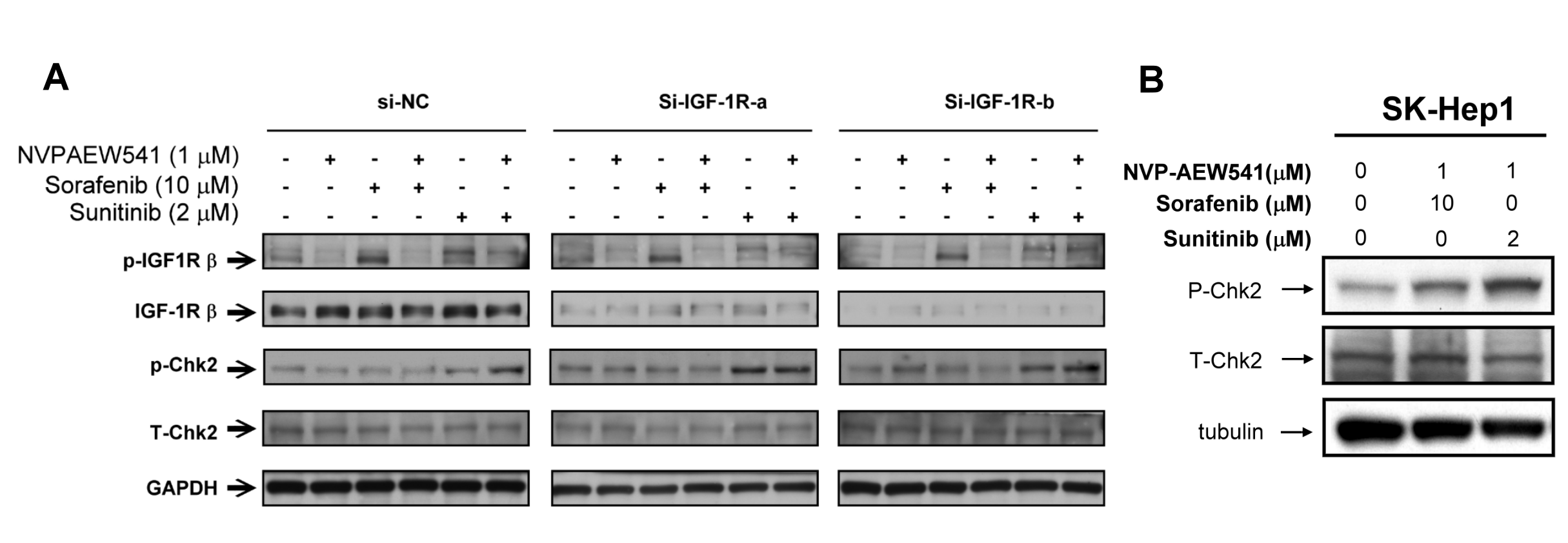

Supplement: Figure S4 — Increased Chk2 phosphorylation by sunitinib plus IGFR inhibition. (DOCX) [file pone.0066589.s004.docx]
