# Supplementary material for: Potentiating the Efficacy of Molecular Targeted Therapy for Hepatocellular Carcinoma by Inhibiting the Insulin-Like Growth Factor Pathway
Source: PLoS One. 2013 Jun 20;8(6):e66589. doi: 10.1371/journal.pone.0066589 (PMC3688529; doi:10.1371/journal.pone.0066589)

Figure S5.

Increased Chk2 phosphorylation contributes to the anti-tumor synergy in SK-hep1 cells between sunitinib and IGFR inhibition.

(A) Median dose effect analysis of synergistic growth inhibitory effects. Growth inhibition was measured by MTT assay. CI was calculated using the CI-isobologram method. CI = 1, additive effect; CI < 1, synergistic effect; CI > 1, antagonistic effect.(B and C) SK-Hep1 cells were transfected with si-Chk2 or scrambled siRNA for 24 hours, and then treated with the indicated drugs. Whole-cell lysates were collected for Western blotting after 48 h drug treatment. The percentages of apoptotic cells were measured by flow cytometry after 72 h drug treatment. **, p < 0.01.


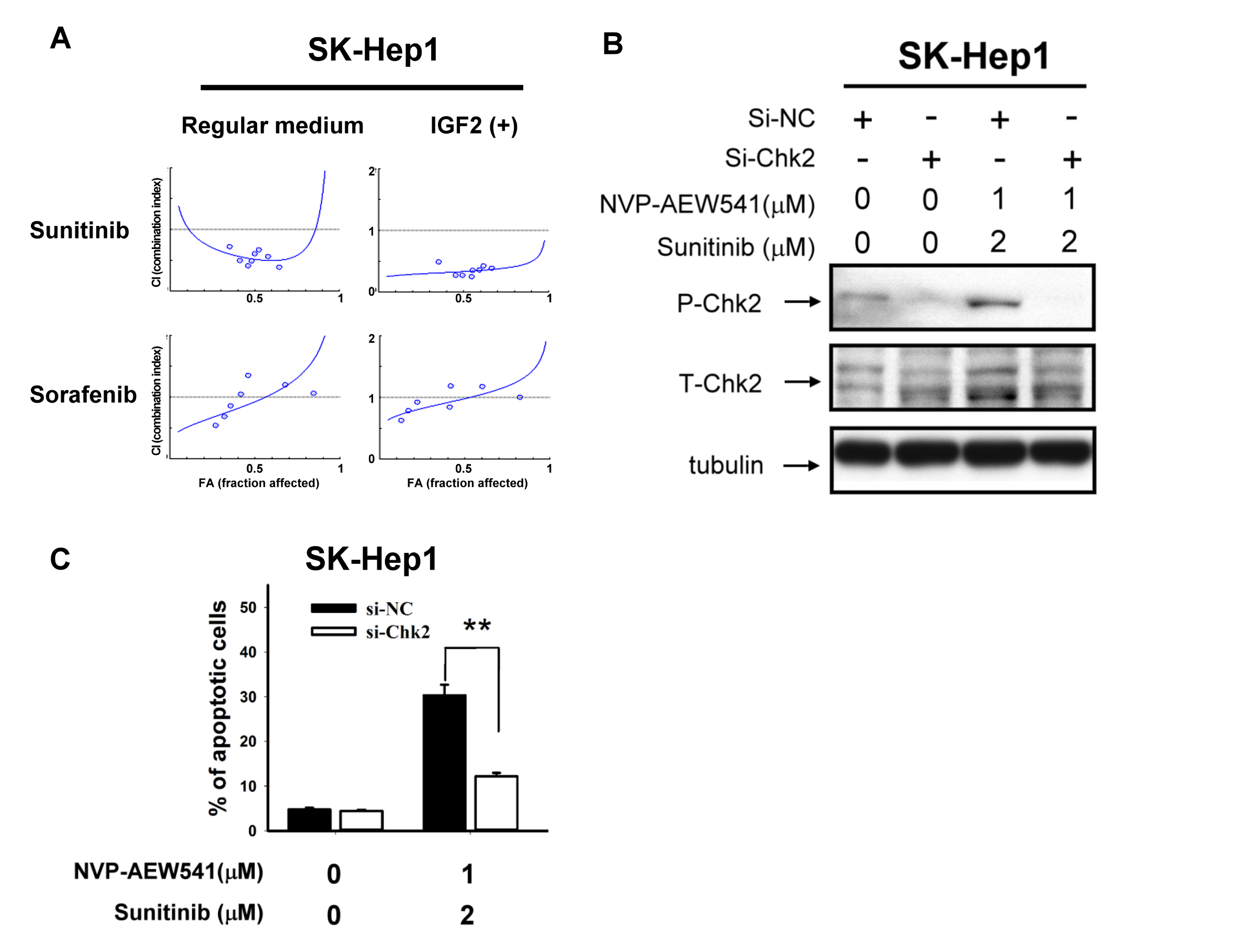

Supplement: Figure S5 — Increased Chk2 phosphorylation contributes to the anti-tumor synergy in SK-hep1 cells between sunitinib and IGFR inhibition. (DOCX) [file pone.0066589.s005.docx]
