# Supplementary material for: Potentiating the Efficacy of Molecular Targeted Therapy for Hepatocellular Carcinoma by Inhibiting the Insulin-Like Growth Factor Pathway
Source: PLoS One. 2013 Jun 20;8(6):e66589. doi: 10.1371/journal.pone.0066589 (PMC3688529; doi:10.1371/journal.pone.0066589)

Figure S6.

The potential anti-angiogenic effects of IGFR inhibition and other MTAs. Hep3B cells were injected subcutaneously into male BALB/c athymic nude mice. Mice were treated daily by gavage as indicated (NVP-30, NVP-AEW541 30 mg/kg/day; Sor-10, sorafenib 10 mg/kg/day; Sun-40, sunitinib 40 mg/kg/day). Tumor microvessel density (MVD) was measured by CD31 immunohistochemical staining (animal number n = 4 in each group). **, P < 0.01, compared with the control (vehicle-treated) group.


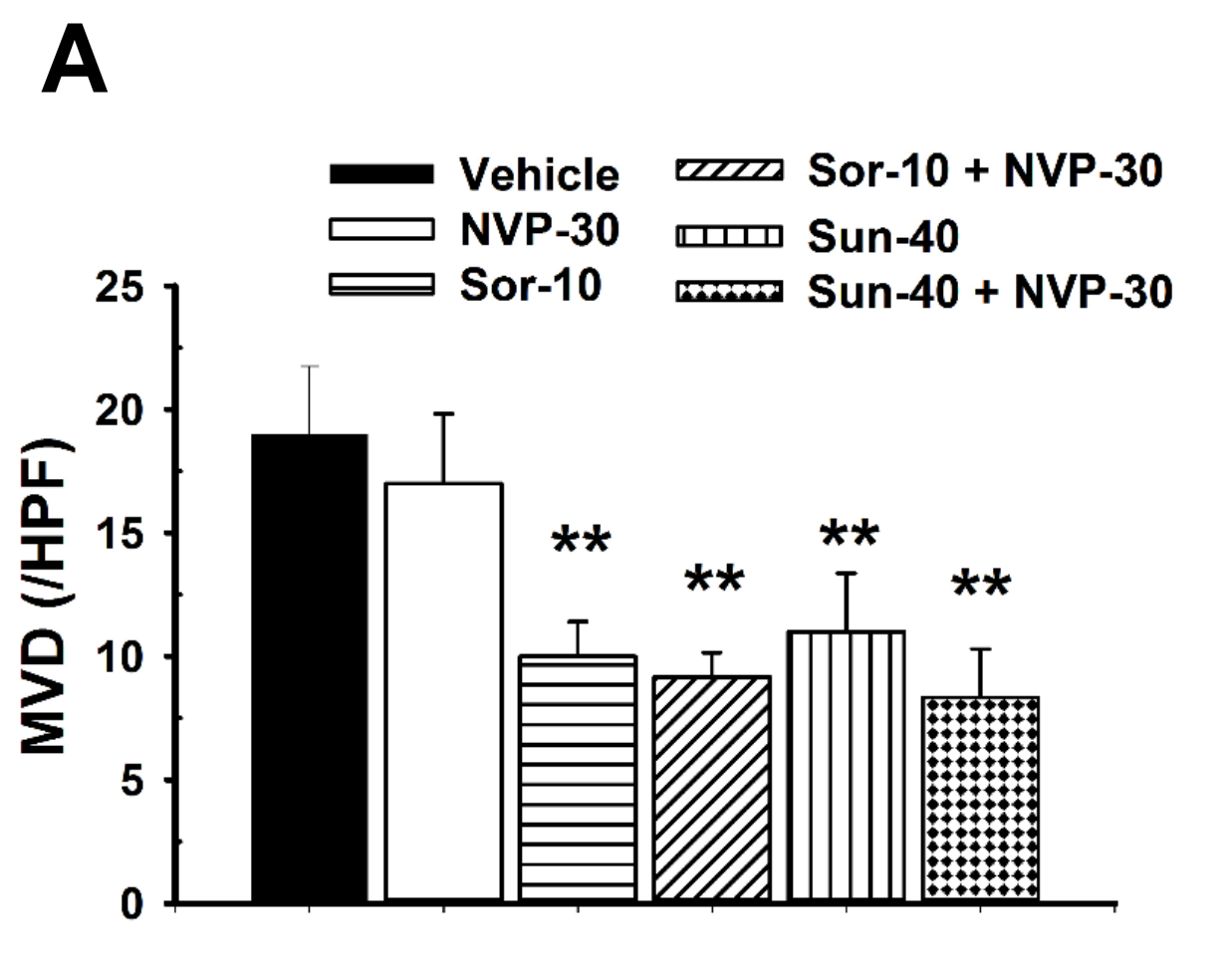

Supplement: Figure S6 — The potential anti-angiogenic effects of IGFR inhibition and other MTAs. (DOCX) [file pone.0066589.s006.docx]
