# Supplementary material for: Potentiating the Efficacy of Molecular Targeted Therapy for Hepatocellular Carcinoma by Inhibiting the Insulin-Like Growth Factor Pathway
Source: PLoS One. 2013 Jun 20;8(6):e66589. doi: 10.1371/journal.pone.0066589 (PMC3688529; doi:10.1371/journal.pone.0066589)
Supplement: Table S1 — Confirmation of the lack of interference with the MTT kit reagents. (DOCX) [file pone.0066589.s007.docx]

Table S1

Confirmation of the lack of interference with the MTT kit reagents. The test compounds were pre-diluted and transferred to 96-well plates by automated liquid handling. 96-well plates were incubated with test compounds for 72 hrs, after which 50 µg/mL of 3-(4,5-Dimethyl-2-thiazolyl)-2,5-dipheny​l-2H-tetrazolium bromide (MTT) was added. After incubation for 4 hrs at 37°C, the medium was discarded and 150 µL of dimethylsulfoxide (DMSO) was added into each well to dissolve the formazan crystals. The absorbance was determined at 540 nm by spectrophometry (DTX 880; Beckman Coulter, Fullerton, CA). As shown in the following table, all the OD values were negligible.
